# Peripheral neutrophils and oxidative stress-associated molecules for predicting the severity of asthma: a cross-sectional study based on multidimensional assessment

**DOI:** 10.3389/fmed.2023.1240253

**Published:** 2023-12-07

**Authors:** Ruolin Mao, Zhilong Jiang, Zhihui Min, Gang Wang, Min Xie, Peng Gao, Lei Zhu, Huayin Li, Zhihong Chen

**Affiliations:** ^1^Department of Respiratory and Critical Care Medicine, Shanghai Institute of Respiratory Disease, Zhongshan Hospital, Fudan University, Shanghai, China; ^2^Department of Respiratory and Critical Care Medicine, Shanghai Chest Hospital, Shanghai Jiao Tong University School of Medicine, Shanghai, China; ^3^Research Center of Zhongshan Hospital, Fudan University, Shanghai, China; ^4^Department of Respiratory and Critical Care Medicine, Clinical Research Center for Respiratory Disease, West China Hospital, Sichuan University, Chengdu, China; ^5^Department of Respiratory and Critical Care Medicine, Tongji Hospital, Tongji Medical College, Huazhong University of Science and Technology, Wuhan, China; ^6^Department of Respiratory Medicine, The Second Affiliated Hospital of Jilin University, Changchun, China; ^7^Department of Respiratory and Critical Care Medicine, Huadong Hospital, Fudan University, Shanghai, China

**Keywords:** severe asthma, neutrophil, oxidative stress, 8-iso-prostaglandin F2alpha, histone deacetylase 2

## Abstract

**Objectives:**

This study aims to explore the relationship between the severity of asthma and neutrophils and related oxidative stress-associated molecules in peripheral blood and induced sputum.

**Methods:**

A total of 67 subjects were included in this study, namely, 25 patients with severe asthma and 42 patients with non-severe asthma. Clinical data, induced sputum and peripheral blood were collected. Lung function and molecules related to oxidative stress in induced sputum and peripheral blood of asthma patients were detected. The relationship between neutrophils and asthma severity was analyzed. HDAC2 mRNA and protein expression levels and HDAC2 activity were also analyzed. Multivariate logistic regression was performed to select statistically significant variables.

**Results:**

The absolute value of neutrophils and percentage of neutrophils were higher in the severe asthma patients. These two values were used to predict the severity of asthma by ROC analysis, with the best cutoff values being 4.55 × 10^9^/L (sensitivity 83.3%, specificity 64.0%) and 55.15% (sensitivity 54.8%, specificity 88.0%). The ROS concentration of neutrophils in the induced sputum samples and the 8-iso-PGF2α concentration in the peripheral blood samples were higher in the severe asthma group (*P* = 0.012; *P* = 0.044), whereas there was reduced HDAC2 protein activity in PBMCs (*P* < 0.001). A logistic equation and a nomogram were created to give a precise prediction of disease severity.

**Conclusion:**

Oxidative stress is increased in severe asthma patients. Peripheral blood neutrophils and 8-iso-PGF2α can be used as biomarkers to predict the severity of asthma. A prediction model was created for evaluating asthma severity.

## 1 Introduction

Severe asthma (SA) patients comprise a subpopulation of patients with asthma who require treatment with medications for Global Initiative for Asthma (GINA) steps 4–5 to maintain disease stability. Although only 5–10% of all asthma patients have SA, it is associated with significant morbidity and mortality and with substantial psychological and socioeconomic burdens (1).

Asthma is a chronic airway inflammatory disease that involves a variety of inflammatory cells and cytokines, such as eosinophils, neutrophils, mast cells, and T cells, which have obvious heterogeneity and complexity (2, 3). Neutrophils are involved in both the inflammatory response and oxidative stress, and their role in the occurrence and development of asthma has received increasing attention in recent years (4, 5). Understanding the relationship between airway inflammation and clinical asthma outcomes in real life is of great importance to better understand the disease and choose the appropriate treatment (6).

Reactive oxygen species (ROS) and reactive nitrogen species (RNS) can be generated during the aggregation and activation of inflammatory cells and play an important role in the occurrence and development of asthma (7, 8). In addition, previous data have demonstrated that an imbalance between oxidant-antioxidant and impaired airway macrophage function is associated with the severity of disease (9).

Accordingly, there is an urgent need to identify the featured characteristics in populations with SA and the relationship between these characteristics and neutrophils and oxidative stress. This study was designed to explore the sociodemographic and clinical characteristics of SA patients and the effects of systemic and airway neutrophils on asthma.

## 2 Material and methods

### 2.1 Study design and subjects

This was a cross-sectional study. Adult subjects (≥ 18 years old) diagnosed with asthma were recruited from the clinics of Zhongshan Hospital, Fudan University, from August 2020 to December 2021.

All the subjects had been previously diagnosed with asthma, and the diagnosis was confirmed by clinicians according to the GINA guidelines based on a history of variable respiratory symptoms and confirmed variable expiratory airway limitation. All the recruited patients were in a stable condition, which was defined as no exacerbation or respiratory tract infection for at least 1 month before enrollment. We excluded subjects who were pregnant or breastfeeding or had other chronic respiratory diseases or chronic unstable diseases of other systems or malignancies.

This study was conducted in accordance with the Declaration of Helsinki. The institutional review board of Zhongshan Hospital, Fudan University reviewed and approved this study (No. 2018-196). All included patients gave written informed consent prior to participation.

### 2.2 Definition and assessment of SA

According to the American Thoracic Society (ATS)/European Respiratory Society (ERS) guidelines (10, 11), SA is defined as asthma that requires treatment with suggested medications for GINA steps 4–5 asthma for the previous year or systemic corticosteroid (CS) use for ≥ 50% of the previous year to prevent uncontrolled asthma or asthma that remains uncontrolled despite this therapy. Based on the severity of asthma, all the included participants were classified into the SA group or the non-severe asthma (NSA) group.

### 2.3 Data collection and clinical assessments

Multidimensional assessments, including sociodemographic characteristics, asthma duration, smoking history, allergen detection results, comorbidities, medication use and acute asthma exacerbation, were performed for all included patients. Asthma control was assessed using the Asthma Control Questionnaire (ACQ) (12), the Mini Asthma Quality of Life Questionnaire (Mini-AQLQ) (13), and the Asthma Control Test (ACT) (14).

The participants also underwent spirometry, FeNO tests, routine blood tests and serum total IgE detection. The FeNO test was performed using a FeNO analyzer (NIOX MINO, Aerocrine AB, Sweden). After the FeNO test, pulmonary function and bronchial dilation tests were performed using spirometry (Master Screen-PFT, Jaeger, Germany). Routine blood tests were performed with a hematology analyzer (Sysmex XE-2100 Fully Automatic Hematology Analyzer, Sysmex, Japan). Serum total IgE was measured by immunoassay (Hitachi 7600 automated biochemistry analyzer, Hitachi, Japan), with a minimum detectable level of IgE of 1.0 IU/mL.

### 2.4 Sputum induction and processing

Sputum induction was performed with routine standard methods. Briefly, subjects underwent spirometry and then gargled and blew their nose. Ten minutes after 100 μg of inhaled albuterol, a 7-min sputum induction was performed using 4.5% saline atomized by an ultrasonic nebulizer (Cumulus, Heyer, Germany). After gargling and blowing the nose again, the subjects coughed sputum into a clean petri dish. If the subjects had no sputum or too little sputum, the previous step was repeated until sufficient qualified specimens were obtained or the total atomization time reached 21 min. Sputum was induced with 0.9% saline for safety if the FEV1%pred was < 40% at baseline, and the procedure was stopped if the FEV1 declined more than 15% versus baseline.

The sputum samples were processed with plug selection and 0.1% dithiothreitol (DTT) treatment within 2 h. Cytospins were prepared using the centrifugation-smear method (SORVALL Stratos, Thermo Fisher Scientific, USA) and stained (May-Grunwald Giemsa), and then cell counting and classification (eosinophils, macrophages, neutrophils, and lymphocytes) were performed independently by two well-trained lab researchers. The eligibility criteria were epithelial cells < 20%.

### 2.5 Definitions of inflammatory phenotype and neutrophil type

Asthma can be classified into four inflammatory phenotypes according to the ratio of neutrophils and eosinophils in qualified induced sputum specimens: eosinophilic type (eosinophils ≥ 3%, neutrophils < 60%), neutrophilic type (eosinophils < 3%, neutrophils ≥ 60%), mixed granulocytic type (eosinophils ≥ 3%, neutrophils ≥ 60%) and paucigranulocytic type (eosinophils < 3%, neutrophils < 60%) (15, 16).

To facilitate the analysis of the effect of airway neutrophils on asthma, neutrophilic and mixed granulocytic types were combined as the neutrophil-dominant type, and eosinophilic and paucigranulocytic types were combined as the non-neutrophil-dominant type.

### 2.6 Oxidative stress biomarker detection

Serum and induced sputum supernatant were collected, and the concentrations of myeloperoxidase (MPO), neutrophil elastase (NE), 8-iso-prostaglandin F2alpha (8-iso-PGF2α) and superoxide dismutase (SOD) were detected by using an ELISA kit (Shanghai Weiao Biotech Co., Ltd., Shanghai, China).

The neutrophil and ROS concentrations of neutrophils in induced sputum were detected by flow cytometry. The neutrophils were labeled with mouse anti-CD66b-PE antibody (BD Pharmingen, USA) and mouse anti-CD11b-FITC antibody (BD Pharmingen, USA). ROS were tested by the CellROX deep red flow cytometry assay kit (Life Technology, USA). Flow cytometry was performed with 405 nm, 488 nm and 633 nm laser wavelengths, and the average fluorescence intensity of ROX Deep Red dye in neutrophils represented the ROS concentration.

### 2.7 Histone deacetylase 2 (HDAC2) expression detection

HDAC2 mRNA expression was detected by qRT–PCR in induced sputum cells and peripheral blood leucocytes. Peripheral blood samples were treated with erythrocyte lysate to obtain peripheral blood leukocytes. Total RNA was extracted from cells by using TRIzol reagent (Invitrogen, USA), and a micro ultraviolet spectrophotometer (Nano Drop 2000, Thermo Fisher Scientific, USA) was used to measure the concentration and purity of total RNA. A real-time PCR System (LightCycler96, Roche, Switzerland) and SYBR Green Master Mix (QIAGEN, Germany) were used for qRT–PCR analysis. GAPDH was used as an internal control for HDAC2. The PCR primers were as follows: HDAC2, forward: 5′-ctgttaattgggctggagga-3′, reverse: 5′-aattcaaggatggcaagcac-3′, and GAPDH, forward: 5′-gcgagatccctccaaaatcaa-3′, reverse: 5′-gttcacacccatgacgaacat-3. The relative expression levels of HDAC2 were calculated by using the 2−△△CT method.

HDAC2 protein expression in peripheral blood mononuclear cells (PBMCs) was detected by Western blot assay. PBMCs were isolated from peripheral blood by using lymphocyte isolation fluid (Dakewe Biotech Co., Ltd., Shenzhen, China). Proteins of whole-cell extracts were extracted from PBMCs by using RIPA buffer supplemented with protease inhibitors (Shanghai Weiao Biotech Co., Ltd., Shanghai, China), and a BCA kit (Shanghai Jingke Chemical Technology Co., Ltd., Shanghai, China) was used to detect the protein concentration. Proteins were separated on an SDS–PAGE gel by electrophoresis. After electrophoresis, proteins were transferred to polyvinylidene fluoride (PVDF) membranes. After incubation with 5% bovine serum albumin, the PVDF membranes were incubated with primary antibodies, namely, rabbit anti-HDAC2 antibody (1:1,800 dilution, Abcam, UK) and mouse anti-GAPDH antibody (1:2,000 dilution, Abcam, UK), at 4°C overnight. Then, the membranes were incubated with horseradish peroxidase-conjugated secondary antibodies for 2 h at room temperature. The bands were detected by using a Supersensitive Enhanced Chemiluminescence Substrate Kit (Shanghai Weiao Biotech Co., Ltd., Shanghai, China). GAPDH was used as an internal control. Bands were visualized with a GelDoc EZ imager (BioRad, USA).

### 2.8 HDAC2 activity detection

Serum was collected, and HDAC2 activity was detected with a human HDAC2 assay kit (ABNOVA, USA).

### 2.9 Statistical analyses

Descriptive analysis of variables is presented as *n* (%) for categorical data, and continuous data are presented as the mean with standard deviations. We compared continuous variables using one-way ANOVA appropriately and categorical variables using chi-square tests among participants of two groups. In addition, *post hoc* Bonferroni comparisons were performed to explore differences between groups, in which the cutoff significance was set at α/*n* (α = 0.05; *n* is the number of comparisons).

To study the influence of neutrophils on asthma severity, we differentiated SA or NSA as an outcome, drew receiver operating characteristic (ROC) curves with the absolute value or percentage of neutrophils as independent variables, and took the maximum Youden index as the basis for determining the cutoff value.

We examined the associations between each variable and asthma severity through univariate logistic regression models. The top 5 variables associated with asthma severity in the univariate analysis were included in multivariate logistic regression models, and a model was established to investigate factors associated with SA. After checking the ROC and calibration curve of the prediction regression model that basically met the requirements, a nomogram model was established to assess the probability of SA.

Linear regression models were established to investigate independent factors associated with neutrophils and each other. Variables associated with neutrophils were included in adjusted multivariate models, and the adjusted rate ratio (aRR) with 95% confidence interval (CI) was calculated. According to the analysis content, age, sex, body mass index (BMI) and smoking status might be regarded as potential confounders of regression analysis.

Data analyses were performed with SPSS 23.0 software (SPSS, Inc., USA) and R 3.4.1 (MathSoft, USA). Two-sided *P* < 0.05 was considered statistically significant.

## 3 Results

### 3.1 SA patients were more likely to develop AE and have more serious pulmonary function injury

Of the 67 participants included in our study, 25 were SA patients, and 42 were NSA patients. In the NSA group, patients controlled their disease with regular or intermittent inhalations, with a maximum therapeutic dose of Seretide 250 (salmeterol/fluticasone 50 ug/250 ug) or Symbicort 160 (budesonide/formoterol 160 ug/4.5 ug) one inhalation at a time, twice a day. No systemic steroids were used. In the SA group, patients regularly used inhaled drugs over a long period of time, the lowest dose was Seretide 500 (salmeterol/fluticasone 50 ug/500 ug) or Symbicort 320 (budesonide/formoterol 320 ug/9 ug) one inhalation at a time, twice a day. A total of 44% (11/25) patients had long-term systemic steroid use, 36% (9/25) patients took montelukast sodium orally on a long-term basis. None of the patients are currently using biologics agents. The sociodemographic and clinical characteristics of patients from the SA and NSA groups are shown in Table 1.

**TABLE 1:** Sociodemographic and clinical characteristics of asthma patients with different severities.

	Severe asthma	Non-severe asthma	χ^2^/t	*P*-value
	*n* = 25	*n* = 42		
Male, *n* (%)	12 (48.00)	22 (52.38)	0.120	0.729
Age, year	58.6 ± 9.41	53.95 ± 13.99	1.452	0.151
BMI, kg/m^2^	24.34 ± 3.62	24.26 ± 3.49	0.078	0.938
Asthma duration, year	27.48 ± 20.13	15.86 ± 16.76	2.505	0.015
**Smoking status, *n* (%)**			1.187	0.552
Never	20 (80.00)	35 (83.33)		
Ever	3 (12.00)	6 (14.29)		
Current	2 (8.00)	1 (2.38)		
**Allergen detection, *n* (%)**			1.908	0.385
Positive	11 (44.0)	15 (35.7)		
Negative	8 (32.0)	10 (23.8)		
Undetected	6 (24.0)	17 (40.5)		
With other allergic diseases, *n* (%)	23 (92.00)	35 (83.33)	1.012	0.314
AE in last year, time	1.32 ± 1.19	0.33 ± 0.84	3.913	<0.001
FVC%pred	75.33 ± 17.02	85.39 ± 23.37	1.813	0.075
FEV1%pred	51.52 ± 19.13	71.72 ± 24.64	3.400	0.001
FEV1/FVC	52.61 ± 11.54	66.10 ± 15.01	4.427	<0.001
DLCO%pred	89.95 ± 22.90	101.21 ± 15.77	2.201	0.032
**Bronchial dilation test, *n* (%)**			2.382	0.497
Positive	8 (32.00)	9 (21.43)		
Probable positive	6 (24.00)	13 (30.95)		
Negative	9 (36.00)	19 (45.24)		
Undetected	2 (8.00)	1 (2.38)		
FeNO, ppb	58.41 ± 44.17	55.73 ± 39.24	0.238	0.813
ACT	18.52 ± 4.07	19.83 ± 4.07	1.258	0.213
ACQ	13.38 ± 5.86	9.93 ± 5.09	2.452	0.017
Mini-AQLQ	70.48 ± 12.80	70.48 ± 14.48	0.001	0.999
Blood Neu, 10^9^/L	5.24 ± 1.87	3.64 ± 1.25	4.151	<0.001
Blood Eos, 10^9^/L	0.27 ± 0.24	0.34 ± 0.31	0.987	0.327
Blood Neu, %	62.93 ± 7.95	54.84 ± 7.57	4.088	<0.001
Blood Eos, %	3.45 ± 2.95	5.46 ± 4.83	1.872	0.066
IgE, IU/ml	326.20 ± 478.46	288.38 ± 550.29	0.281	0.779
Blood 8-iso-PGF2α, pmol/ml	162.41 ± 307.32	55.14 ± 45.47	2.056	0.044
Blood NE, pg/ml	1,877.35 ± 1,920.51	1,868.83 ± 1,605.74	0.019	0.985
Blood SOD, ng/ml	133.39 ± 25.64	114.35 ± 33.03	2.400	0.019
Blood MPO, ng/ml	120.42 ± 36.65	148.63 ± 35.75	2.985	0.004

There was no significant difference in sex, age or BMI between the two groups, but the asthma duration in the SA group was significantly longer than that in the NSA group (27.48 ± 20.13 years vs. 15.86 ± 16.76 years, *P* = 0.015). The SA group patients were also more likely to develop acute exacerbation (AE) in the last year (1.32 ± 1.19 times vs. 0.33 ± 0.84 times, *P* < 0.001). The vast majority of asthma patients in both groups had never smoked (80.00 vs. 83.33%) and had other allergic diseases (92.00 vs. 83.33%).

The pulmonary function test (PFT) results for the two groups showed significant differences in FEV1%pred (51.52 ± 19.13% vs. 71.72 ± 24.64%, *P* = 0.001), FEV1/FVC (52.61 ± 11.54% vs. 66.10 ± 15.01%, *P* < 0.001) and DLCO%pred (89.95 ± 22.90% vs. 101.21 ± 15.77%, *P* = 0.032). The results showed that the impaired ventilation and diffusion function of the SA patients were significantly more serious than those of the NSA patients. However, there were no significant differences in the FeNO results (*P* > 0.05).

Unlike the ACQ results (13.38 ± 5.86 vs. 9.93 ± 5.09, *P* = 0.017), there was no significant difference in the ACT and Mini-AQLQ results.

### 3.2 Neutrophils in the peripheral blood of the SA patients were significantly increased

**FIGURE 1:**
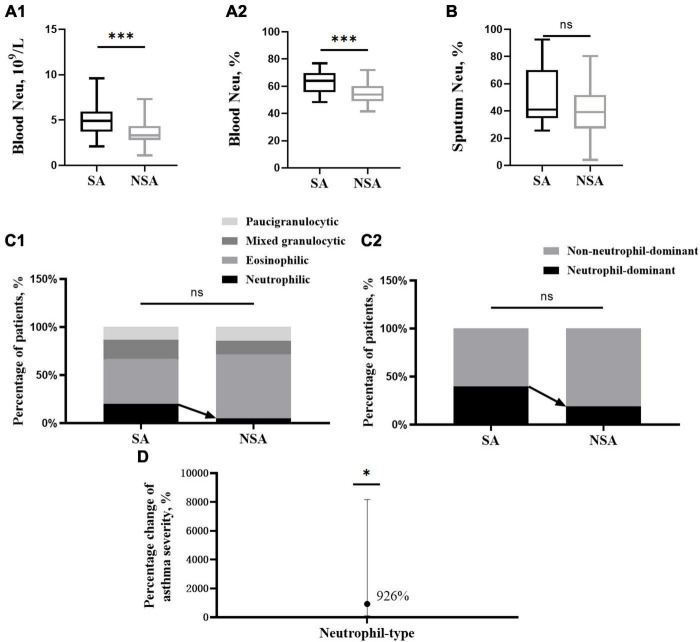
Differences in neutrophils and associated phenotypes in patients with asthma of different severities. This figure shows *t*-test results of neutrophils in peripheral blood **(A1,A2)** and induced sputum **(B)** and the percentage of inflammatory phenotype **(C1)** and neutrophil type **(C2)** between the severe (black block) and non-severe (gray block) asthma groups. The effect of neutrophil type on asthma severity **(D)**. ns: *P* > 0.05, *: *P* < 0.05, ***: *P* < 0.001.

Blood samples were collected from 25 SA patients and 42 NSA patients, and the absolute value (5.24 ± 1.87 10^9^/L vs. 3.64 ± 1.25 10^9^/L, *P* < 0.001) and percentage (62.93 ± 7.95% vs. 54.84 ± 7.57%, *P* < 0.001) of neutrophils in the SA group were significantly higher than those in the NSA group, and there was no significant difference in eosinophil levels. The results are shown in Figures 1A1, A2 and Table 1.

### 3.3 Neutrophil-dominated airway inflammation in sputum was more likely to present as SA

Induced sputum collection was attempted in all the participants, and samples were obtained from 19 SA patients and 23 NSA patients. After sputum quality screening, the induced sputum samples from 15 SA patients and 21 NSA patients met the eligibility criteria (a percentage of epithelial cells < 20%).

The neutrophils in induced sputum were slightly higher in patients in the SA group (51.51 ± 22.09% vs. 40.04 ± 18.37%, *P* = 0.108). After classifying the inflammatory phenotype based on sputum cell counts, the proportion of the neutrophilic type in the SA group was higher than that in the NSA group (20.00 vs. 4.76%, *P* = 0.456), and the proportion of the neutrophil-dominant type in the SA group was also higher (40.00 vs. 19.05%, *P* = 0.166). Due to the relatively small number of qualified sputum samples, the results were not significantly different. The results are shown in Figures 1B, C.

After adjusting for the four covariables of age, sex, BMI and smoking status, the neutrophil-dominant type and asthma severity were analyzed by multivariate logistic regression, and a positive relative risk was found (RR = 9.26, CI [1.05, 81.70], *P* = 0.045). This means that the risk of SA was 9.26 times higher in those with neutrophil-dominant asthma than in those with non-neutrophil-dominant asthma. The results are shown in Figure 1D.

### 3.4 High level of neutrophils in peripheral blood can predict SA

**FIGURE 2:**
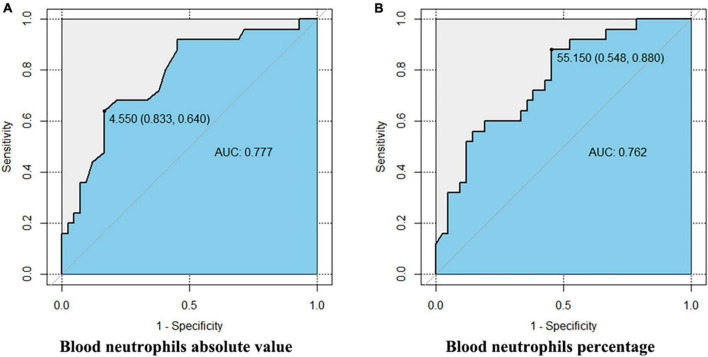
The ROC of peripheral blood neutrophils predicting severe asthma. This figure shows the Delong test results of the absolute value **(A)** and percentage **(B)** of peripheral blood neutrophils and severe asthma. The AUCs of the two are 0.777 (CI [0.660, 0.893]) and 0.762 (CI [0.646, 0.879]), and the best cutoff values are 4.55 × 10^9^/L (sensitivity 83.3%, specificity 64.0%) and 55.15% (sensitivity 54.8%, specificity 88.0%), respectively.

Receiver operating characteristic (ROC) analysis showed that both the absolute value and percentage of neutrophils were predictive parameters for SA. The AUCs of the two were 0.777 (CI [0.660, 0.893]) and 0.762 (CI [0.646, 0.879]), and the best cutoff values were 4.55 × 10^9^/L (sensitivity 83.3%, specificity 64.0%) and 55.15% (sensitivity 54.8%, specificity 88.0%), respectively. The results are shown in Figure 2.

### 3.5 Composite clinical indexes, including blood neutrophils, predict the probability of SA

**FIGURE 3:**
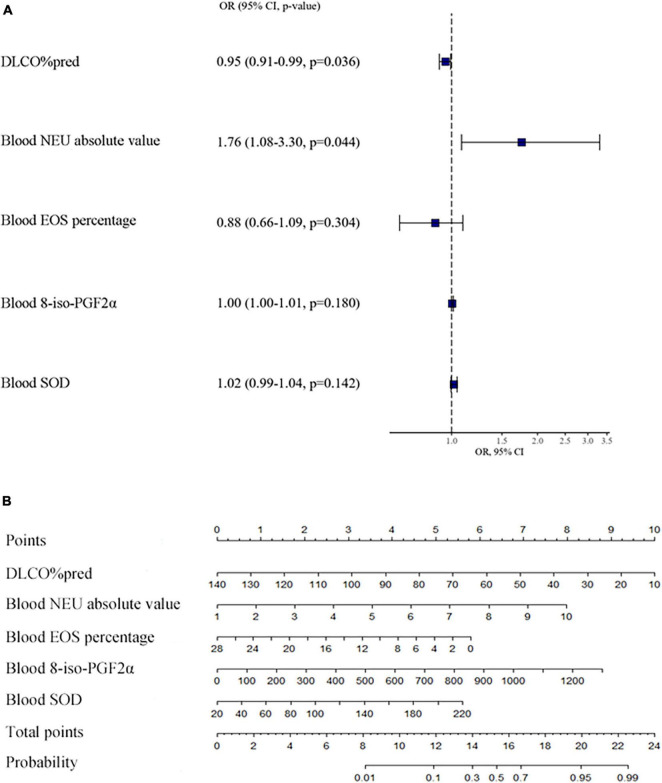
Influence of various factors on asthma severity and the nomogram for assessing the probability of severe asthma. This figure **(A)** shows the multivariate logistic regression model of factors on asthma severity. This figure **(B)** shows the nomogram of the multivariate logistic regression model of the probability of severe asthma. We can see a total of 8 horizontal lines with scales, the first one is “points.” We could evaluate the variables of horizontal lines 2-6 according to patient’s situation, and draw a vertical line upward, respectively, to the first horizontal line, and then get the points of each item. All the points are then added together to get the “total points,” which is marked on the seventh horizontal line. Finally, draw a vertical line down to the eighth horizontal line, to estimate the “probability” that the patient will have severe asthma. DLCO: carbon monoxide diffusion capacity, NEU: neutrophil, EOS: eosinophil, 8-iso-PGF2α:8-iso-prostaglandin F2alpha, SOD: superoxide dismutase.

By multivariate logistic regression, we found that decreased DLCO%pred and an increased absolute value of neutrophils in blood were statistically significant for predicting SA, and the results are shown in Figure 3A. Then, we obtained the multivariate logistic regression model: Ln[pr(Severe Asthma)/1-pr(Severe Asthma)] = −0.133–0.049 × DLCO%pred + 0.565 × blood absolute value of neutrophils −0.132 × blood percentage of eosinophils + 0.004 × blood 8-iso-PGF2α + 0.018 × blood SOD. The ROC of the prediction regression model was 0.8612 (CI [0.7546, 0.9678]). The calibration curve also basically agreed with the statistical requirements. Then, a nomogram for assessing the probability of SA was developed and is shown in Figure 3B.

### 3.6 Oxidative stress damage was more severe in SA patients

**FIGURE 4:**
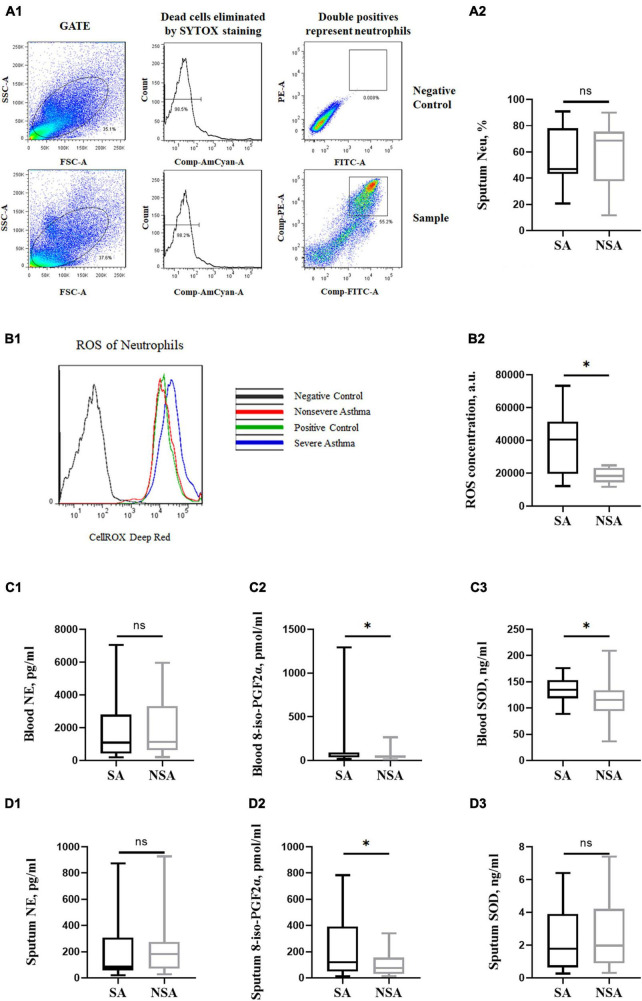
Oxidative stress of patients with asthma of different severities. This figure shows the flow cytometry of neutrophils and ROS concentration in induced sputum **(A1,B1)**. *T*-test results of neutrophils and ROS concentrations **(A2,B2)** and NE, 8-iso-PGF2α and SOD concentrations in peripheral blood **(C1–C3)** and induced sputum **(D1–D3)** between the severe (black block) and non-severe (gray block) asthma groups. ns: *P* > 0.05, *: *P* < 0.05.

There was no significant difference in induced sputum neutrophils between the SA and NSA groups, but the ROS concentration of neutrophils in the SA group was significantly higher (39,331.50 ± 20,101.99 a.u. vs. 18,357.25 ± 4,646.68 a.u., *P* = 0.012). The results are shown in Figures 4A, B.

The 8-iso-PGF2α concentration of neutrophils in the SA group was higher in both peripheral blood (162.41 ± 307.32 pmol/ml vs. 55.14 ± 45.47 pmol/ml, *P* = 0.044) and induced sputum (239.92 ± 234.13 pmol/ml vs. 106.39 ± 97.78 pmol/ml, *P* = 0.022). In addition, the SOD concentration in the peripheral blood of SA patients was also higher (133.39 ± 25.64 ng/ml vs. 114.35 ± 33.03 ng/ml, *P* = 0.019). There was no significant difference in the NE concentration between the two groups. The results are shown in Figures 4C, D.

### 3.7 MPO was lower in SA patients

The MPO concentration in the SA group was lower in peripheral blood (120.42 ± 36.65 ng/ml vs. 148.63 ± 35.75 ng/ml, *P* = 0.004), and there was no significant difference in induced sputum (5.47 ± 2.61 ng/ml vs. 4.98 ± 2.72 ng/ml, *P* = 0.579). MPO concentration in peripheral blood was significantly positively correlated with SOD concentration (β = 0.470, *P* = 0.002), while the correlation between the two was not obvious in induced sputum. The neutrophils in peripheral blood or induced sputum increased, but the MPO concentration in blood decreased (*P* < 0.001, *P* = 0.013), no significant relationship with MPO concentration in induced sputum. And the MPO concentration in peripheral blood was negatively correlated with NE concentration (β = −0.004, *P* = 0.041). MPO concentration in induced sputum was positively correlated with 8-iso-PGF2α concentration (β = 31.43, *P* = 0.003), and no significant relationship in peripheral blood.

### 3.8 HDAC2 activity was impaired in SA patients

**FIGURE 5:**
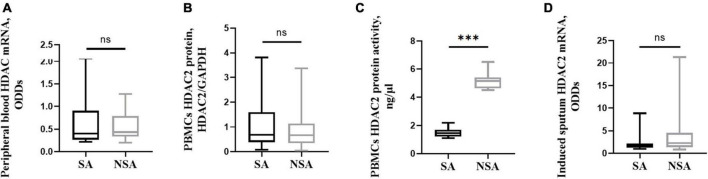
HDAC2 expression and activity in patients with asthma of different severities. This figure shows *t*-test results of the expression of peripheral blood HDAC2 mRNA **(A)**, and PBMCs HDAC2 protein **(B)**, PBMCs HDAC2 protein activity **(C)**, as well as the induced sputum HDAC2 mRNA **(D)** between the severe (black block) and non-severe (gray block) asthma group. ns: *P* > 0.05, ***: *P* < 0.001. PBMCs: peripheral blood mononuclear cells.

Peripheral blood cells and induced sputum cells were collected and assessed for HDAC2 mRNA via qRT–PCR. There was no significant difference in gene expression between the two groups (*P* > 0.05). HDAC2 protein expression and activity were detected in PBMCs. The results showed that the activity of HDAC2 in SA patients was significantly lower than that in NSA patients (1.517 ± 0.338 vs. 5.133 ± 0.319, *P* < 0.001). The results are shown in Figure 5.

## 4 Discussion

Airway inflammation is a common pathological feature of asthma patients. In addition to eosinophils, the role of neutrophils in the occurrence and development of asthma has received increasing attention in recent years (17). Some studies have found that neutrophilic inflammation in the acute phase of asthma can be observed and is associated with glucocorticoid resistance in SA patients and that induced sputum neutrophil levels increase in some patients with chronic persistent asthma (18–23). The neutrophils in induced sputum are also correlated with the degree of airway obstruction and severity of asthma (18, 24).

Neutrophils contain and release a powerful arsenal of mediators, including several granular enzymes, ROS and neutrophil extracellular traps (25). The occurrence and development of asthma is related to Th2 inflammation, and studies on human and animal respiratory virus infections have confirmed that neutrophils can promote Th2 inflammation through DNA extracellular traps (26–28).

Peripheral blood cell counting is a regular clinical lab test that is simple and non-invasive and can be widely carried out in primary medical institutions. Based on the absolute value of eosinophils and neutrophils in peripheral blood, Nadif et al. (29) classified asthma patients into 4 categories and found that a substantial number of asthma patients (56.2%) had the eosinophil-low pattern (< 250 eosinophils/mm^3^). Although some studies have confirmed that neutrophils are significantly active in the peripheral blood of SA patients (30) and that the autophagy and extracellular DNA traps of peripheral blood neutrophils could enhance asthma severity by damaging the airway epithelium and triggering inflammatory responses of airway epithelial cells and peripheral blood eosinophils (31), the role of neutrophils in the peripheral blood in the diagnosis of asthma and the judgment of its severity have not yet been determined.

In our study, we found that peripheral blood neutrophils can be used as biomarkers for predicting SA, and it is believed that asthma patients with absolute values greater than 4.55 × 10^9^/L and proportions higher than 55.15% are likely to have SA, with a sensitivity of 83.3%/54.8% and specificity of 64.0%/88.0%, respectively. In addition, a multivariate logistic regression was performed by introducing the variables selected in the univariate regression model, and the statistically significant variables, such as peripheral blood cells, lung function, plasma oxidative stress biomarker 8-iso-PGF2α and SOD, were identified. Finally, a logistic equation and a nomogram were created to give a precise prediction of disease severity for a given patient. This has not been reported in previous studies.

The concentration of the lipid membrane peroxidation damage product 8-iso-PGF2α can reflect the level of oxidative stress injury. The peroxidation reaction of membrane lipids can lead to fatty acid chain rupture, aggravate airway inflammation and cause tissue damage and morphological changes (32). Lipid peroxidation is also believed to be closely related to asthma, and increased concentrations of 8-iso-PGF2α and its congeners have been found in the exhaled condensate, induced sputum, peripheral blood and urine of asthma patients (33–36). Its concentration in the blood of asthma patients increased significantly in the acute exacerbation stage of asthma and decreased in the remission stage and was significantly higher than that of healthy people (37). In our study, this result is consistent with previous literature, that is, both in peripheral blood and induced sputum, the level of 8-iso-PGF2α in patients with SA was significantly increased.

Interestingly, an antioxidant substance, SOD, increased with the increase in oxidative damage products. SOD is the main enzymatic antioxidant, including three isozymes, among which extracellular SOD is the main SOD in the extracellular range and is highly expressed in lung tissues (37). We were also doubtful about the increase of MPO in NSA group, and it was negatively correlated with neutrophils in peripheral blood. To verify whether there is an enhanced oxidation reaction in patients with SA, ROS in induced sputum were directly measured by flow cytometry. The results showed that ROS in the SA group was significantly increased, indicating that an enhanced oxidation reaction did exist.

Antioxidation is a cellular and cellular product defense system established by the body to resist oxidative substances and prevent bodily damage. Some studies have found that the concentration of total glutathione (including glutathione and oxidized glutathione) in induced sputum and alveolar bronchial lavage in NSA patients is significantly higher than that in healthy people (38, 39). Ammar et al. (40) also found that increased SOD activity was one predictor of poorly controlled asthma. Based on the above, we believe that there is a compensatory effect of antioxidant activity in asthma patients and that when oxidative activity is increased, the antioxidant capacity is increased, even beyond the baseline capacity. However, the compensatory protection of antioxidant activity did not surpass the oxidative damage, so the increase in the oxidative stress product 8-iso-PGF2α was more pronounced. The mechanisms that determine the compensatory increase in antioxidant activity in asthma patients still need further study.

Myeloperoxidase (MPO) is often considered an indicator of neutrophil activity. The release of MPO during the activation of neutrophils is related to airway hyperreactivity (41), which can catalyze the reaction between hydrogen peroxide and chloride to produce hypochlorous acid with higher activity and stronger toxicity, which is considered to be a significant marker of inflammatory response (42), while nitrite formed by nitric oxide oxidation is an important substrate for MPO production (43). However, according to our results, we believe that MPO concentration cannot be used to determine the activation intensity of neutrophils in the body. A similar view has been found in studies of childhood asthma (44), which suggest that serum MPO is not involved in the assessment of inflammatory processes in childhood asthma, and the measurement of serum MPO appears to have no role in assessing the involvement of neutrophils in asthma.

As a protease, HDAC2 plays an important role in the modification of chromosome structure and regulation of gene expression and is related to the deacetylation of a variety of transcription factors, receptors and histones. HDAC2 is widely expressed and exists in the nucleus, and its decreased activity is correlated with hormone resistance (45). Oxidative stress can affect the expression and activity of HDAC2 protein in a variety of ways, resulting in hormone resistance (46), which may adversely affect the treatment of asthma. In SA patients, HDAC2 activity and expression are decreased, which may lead to a decreased glucocorticoid treatment effect and an enhanced inflammatory response (47, 48). In our study, HDAC2 activity was significantly reduced in the SA group. The mRNA expression of HDAC2 both in peripheral blood and in sputum showed a decreasing trend (due to the lower sample size, there was no statistical significance). These results suggest that HDAC2 expression may be impaired in asthma patients with neutrophilic and mixed granulocytic inflammatory phenotypes, leading to insensitivity to glucocorticoid therapy. They also suggest that the diagnosis and treatment of asthma, especially neutrophil-dominant asthma, need further exploration from the aspect of HDAC2 impairment. Until now, roxithromycin, 1,25-dihydroxyvitamin D3, clarithromycin and other drugs have been proven to be beneficial to improve the activity of HDAC2 in asthma animal experiments (49–51), which may become a new choice for asthma treatment in the future.

Inevitably, there are many biomarkers associated with neutrophilic asthma that we did not detect at this time, of which interleukin (IL)-17 is currently receiving a lot of attention. Th17 cells/IL-17 plays an important role in host defense and hyperimmune responses against pathogenic bacteria accompanied by the recruitment of neutrophils (52). Th17-associated inflammation usually contributes to the neutrophilic phenotype asthma, which is often characterized by greater severity, airflow obstruction, and steroid resistance (19, 52). IL-17-related cytokines expression was amplified in bronchial/nasal mucosa of neutrophilic asthma prone to exacerbation, suggesting a pathogenic role of IL-17F infrequent exacerbators (53). Animal models and human clinical studies have found that IL-17 exerts these effects through a variety of pathways. Östling et al. (54) found the activation of thromboxane B2 pathway in IL-17-high asthma patients. Hong et al. (55) found that the blood lipocalin-2 (LCN2) and serum amyloid A (SAA) levels may be associated with a type 17 asthma subtype, which are steroid-resistant IL-17A target genes in airway cells, and IL-17A–Act1/CEBPB axis is an important regulatory mechanism of LCN2 and SAA. Although Nascimento et al. (56) showed that treatment with IL-17A antibody contributed to the control of Th1/Th2/Th17 inflammation, chemokine expression, extracellular matrix remodeling, and oxidative stress in a murine model of lipopolysaccharide-exacerbated asthma. However, two clinical trials of humanized anti-IL-17A monoclonal antibodies, secukinumab and CJM112 failed to improve asthmatic symptoms in severe asthma patient (57). What’s more, the studies of brodalumab, a humanized monoclonal antibody that binds to IL-17RA, was stopped because of the relative lack of efficacy in the initial asthma clinical trial and a questionable safety issue (58). These results highlight that studies on the association between ILA-17 and neutrophil-dominant phenotype of severe asthma still need to be continued, which will be one of our future research directions.

## 5 Conclusion

In conclusion, our research suggests a role for neutrophilic-type airway inflammation in the pathogenesis of SA. The relationship among infiltrating neutrophils, oxidative stress damage and dysfunctional HDAC2 might contribute to glucocorticoid resistance. A prediction model for evaluating disease severity based on multidimensional clinical variables has been created, which does not require observing patients’ status for a long time. Because of the complexity and heterogeneity of the pathogenesis of SA, the underlying mechanisms involved in neutrophilic-type SA need further study.

## Data availability statement

The raw data supporting the conclusions of this article will be made available by the authors, without undue reservation.

## Ethics statement

The studies involving humans were approved by the Institutional Review Board of Zhongshan Hospital, Fudan University. The studies were conducted in accordance with the local legislation and institutional requirements. The participants provided their written informed consent to participate in this study.

## Author contributions

ZC and HL conceived the writing structure. RM, ZJ, and ZM enrolled the patients and collected the raw data. RM, GW, MX, PG, and LZ reviewed or analyzed the data statistically. RM, ZC, and HL wrote and modified the manuscript. All authors contributed to the article and approved the submitted version.

## References

[1] Wang E Wechsler ME Tran TN Heaney LG Jones RC Menzies-Gow AN Characterization of severe asthma worldwide: data from the international severe asthma registry. *Chest.* (2020) 157:790–804. 31785254 10.1016/j.chest.2019.10.053

[2] Chung KF. Asthma phenotyping: a necessity for improved therapeutic precision and new targeted therapies. *J Intern Med.* (2016) 279:192–204. 10.1111/joim.12382 26076339

[3] Hoffmann F Ender F Schmudde I Lewkowich IP Köhl J König P Origin, localization, and immunoregulatory properties of pulmonary phagocytes in allergic asthma. *Front Immunol.* (2016) 7:107. 10.3389/fimmu.2016.00107 27047494 PMC4803735

[4] Seys SF Lokwani R Simpson JL Bullens DM. New insights in neutrophilic asthma. *Curr Opin Pulm Med.* (2019) 25:113–20. 30422895 10.1097/MCP.0000000000000543

[5] Volder JD Vereecke L Joos G Maes T. Targeting neutrophils in asthma: A therapeutic opportunity? *Biochem Pharmacol.* (2020) 182:114292. 10.1016/j.bcp.2020.114292 33080186

[6] Louis RE Schleich FN. Granulocytic airway inflammation and clinical asthma outcomes. *Am J Respir Crit Care Med.* (2021) 203:797–9. 33606963 10.1164/rccm.202102-0356ED PMC8017566

[7] Zuo L Otenbaker NP Rose BA Salisbury KS. Molecular mechanisms of reactive oxygen species-related pulmonary inflammation and asthma. *Mol Immunol.* (2013) 56:57–63. 10.1016/j.molimm.2013.04.002 23665383

[8] Liu X Chen Z. The pathophysiological role of mitochondrial oxidative stress in lung diseases. *J Transl Med.* (2017) 15:207. 29029603 10.1186/s12967-017-1306-5 PMC5640915

[9] Groot LE Veen TA Martinez FO Hamann J Lutter R Melgert BN. Oxidative stress and macrophages: driving forces behind exacerbations of asthma and chronic obstructive pulmonary disease? *Am J Physiol Lung Cell Mol Physiol.* (2019) 316:L369–84. 10.1152/ajplung.00456.2018 30520687

[10] Chung KF Wenzel SE Brozek JL Bush A Castro M Sterk PJ International ERS/ATS guidelines on definition, evaluation and treatment of severe asthma. *Eur Respir J.* (2014) 43:343–73. 24337046 10.1183/09031936.00202013

[11] Holguin F Cardet JC Chung KF Diver S Ferreira D Fitzpatrick A Management of severe asthma: a European Respiratory Society/American Thoracic Society guideline. *Eur Respir J.* (2020) 55:1900588. 31558662 10.1183/13993003.00588-2019

[12] Juniper EF Svensson K Mörk A Ståhl E. Measurement properties and interpretation of three shortened versions of the asthma control questionnaire. *Respir Med.* (2005) 99:553–8. 10.1016/j.rmed.2004.10.008 15823451

[13] Juniper EF Guyatt GH Cox FM Ferrie PJ King DR. Development and validation of the mini asthma quality of life questionnaire. *Eur Respir J.* (1999) 14:32–8. 10489826 10.1034/j.1399-3003.1999.14a08.x

[14] Nathan RA Sorkness CA Kosinski M Schatz M Li JT Marcus P Development of the asthma control test: a survey for assessing asthma control. *J Allergy Clin Immunol.* (2004) 113:59–65. 10.1016/j.jaci.2003.09.008 14713908

[15] Ntontsi P Loukides S Bakakos P Kostikas K Papatheodorou G Papathanassiou E Clinical, functional and inflammatory characteristics in patients with paucigranulocytic stable asthma: Comparison with different sputum phenotypes. *Allergy.* (2017) 72:1761–7. 28407269 10.1111/all.13184

[16] Kimura H Suzuki M Konno S Romero D Sanz V López-Carrasco V Sputum periostin in patients with different severe asthma phenotypes. *Allergy.* (2015) 70:884–5. 26081263 10.1111/all.12639

[17] Bruijnzeel PL Uddin M Koenderman L. Targeting neutrophilic inflammation in severe neutrophilic asthma: can we target the disease-relevant neutrophil phenotype? *J Leukoc Biol.* (2015) 98:549–56. 10.1189/jlb.3VMR1214-600RR 25977288

[18] Ray A Kolls JK. Neutrophilic inflammation in asthma and association with disease severity. *Trends Immunol.* (2017) 38:942–54. 28784414 10.1016/j.it.2017.07.003 PMC5711587

[19] Nabe T. Steroid-Resistant Asthma and Neutrophils. *Biol Pharm Bull.* (2020) 43:31–5. 31902928 10.1248/bpb.b19-00095

[20] Su M Lin W Tsai C Chiang B Yang Y Lin Y Childhood asthma clusters reveal neutrophil-predominant phenotype with distinct gene expression. *Allergy.* (2018) 73:2024–32. 10.1111/all.13439 29574758

[21] Nair P Prabhavalkar KS. Neutrophilic asthma and potentially related target therapies. *Curr Drug Targets.* (2020) 21:374–88. 31660822 10.2174/1389450120666191011162526

[22] Naseem A Liaqat J Zaidi SB Iftikhar R. Sputum neutrophilia in severe persistent asthmatics. *J Coll Physicians Surg Pak.* (2014) 24:420–3. 24953917

[23] Bi J Min Z Yuan H Jiang Z Mao R Zhu T PI3K inhibitor treatment ameliorates the glucocorticoid insensitivity of PBMCs in severe asthma. *Clin Transl Med.* (2020) 9:22. 10.1186/s40169-020-0262-5 32112175 PMC7048898

[24] Farah CS Keulers LA Hardaker KM Peters MJ Berend N Postma DS Association between peripheral airway function and neutrophilic inflammation in asthma. *Respirology.* (2015) 20:975–81. 25952106 10.1111/resp.12550

[25] Varricchi G Modestino L Poto R Cristinziano L Gentile L Postiglione L Neutrophil extracellular traps and neutrophil-derived mediators as possible biomarkers in bronchial asthma. *Clin Exp Med.* (2021) 22:285–300. 10.1007/s10238-021-00750-8 34342773 PMC9110438

[26] Cortjens B Boer OJ Jong R d Antonis AF Piñeros YS Lutter R Neutrophil extracellular traps cause airway obstruction during respiratory syncytial virus disease. *J Pathol.* (2016) 238:401–11. 26468056 10.1002/path.4660

[27] Chen X Li Y Qin L He R Hu C. Neutrophil extracellular trapping network promotes the pathogenesis of neutrophil-associated asthma through macrophages. *Immunol Invest.* (2021) 50:544–61. 10.1080/08820139.2020.1778720 32552227

[28] Toussaint M Jackson DJ Swieboda D Guedán A Tsourouktsoglou T Ching YM Host DNA released by NETosis promotes rhinovirus-induced type-2 allergic asthma exacerbation. *Nat Med.* (2017) 23:681–91. 28459437 10.1038/nm.4332 PMC5821220

[29] Nadif R Siroux V Oryszczyn M Ravault C Pison C Pin I Heterogeneity of asthma according to blood inflammatory patterns. *Thorax.* (2009) 64:374–80. 19131450 10.1136/thx.2008.103069

[30] Mann BS Chung KF. Blood neutrophil activation markers in severe asthma: lack of inhibition by prednisolone therapy. *Respir Res.* (2006) 7:59. 10.1186/1465-9921-7-59 16600024 PMC1458332

[31] Pham DL Ban G Kim S Shin YS Ye Y Chwae Y Neutrophil autophagy and extracellular DNA traps contribute to airway inflammation in severe asthma. *Clin Exp Allergy.* (2017) 47:57–70. 10.1111/cea.12859 27883241

[32] Sugiura H Ichinose M. Oxidative and nitrative stress in bronchial asthma. *Antioxid Redox Signal.* (2008) 10:785–97. 18177234 10.1089/ars.2007.1937

[33] Balanz SC Aragonés AM Mir JC Ramírez JB Iváñez RN Soriano AN Leukotriene B4 and 8-isoprostane in exhaled breath condensate of children with episodic and persistent asthma. *J Investig Allergol Clin Immunol.* (2010) 20:237–43. 20635789

[34] Mak JC Ho SP Ho AS Law BK Cheung AH Ho JC Sustained elevation of systemic oxidative stress and inflammation in exacerbation and remission of asthma. *ISRN Allergy.* (2013) 2013:561831. 10.1155/2013/561831 24073339 PMC3773380

[35] Fitzpatrick AM Jones DP Brown LA. Glutathione redox control of asthma: from molecular mechanisms to therapeutic opportunities. *Antioxid Redox Signal.* (2012) 17:375–408. 10.1089/ars.2011.4198 22304503 PMC3353819

[36] Wedes SH Khatri SB Zhang R Wu W Comhair SA Wenzel S Noninvasive markers of airway inflammation in asthma. *Clin Transl Sci.* (2009) 2:112–7. 20234847 10.1111/j.1752-8062.2009.00095.x PMC2838203

[37] Roman J Zhu J Ritzenthaler JD Zelko IN. Epigenetic regulation of EC-SOD expression in aging lung fibroblasts: Role of histone acetylation. *Free Radic Biol Med.* (2017) 112:212–23. 10.1016/j.freeradbiomed.2017.07.028 28757400

[38] Smith LJ Houston M Anderson J. Increased levels of glutathione in bronchoalveolar lavage fluid from patients with asthma. *Am Rev Respir Dis.* (1993) 147:1461–4. 8503557 10.1164/ajrccm/147.6_Pt_1.1461

[39] Beier J Beeh KM Semmler D Beike N Buhl R. Increased concentrations of glutathione in induced sputum of patients with mild or moderate allergic asthma. *Ann Allergy Asthma Immunol.* (2004) 92:459–63. 15104199 10.1016/S1081-1206(10)61783-8

[40] Ammar M Bahloul N Amri O Omri R Ghozzi H Kammoun S Oxidative stress in patients with asthma and its relation to uncontrolled asthma. *J Clin Lab Anal.* (2022) 2022:e24345. 10.1002/jcla.24345 PMC9102642 35318723

[41] Pirogov A Prikhodko A Zinov Ev S Borodin EA. Specific features of bronchial inflammation in asthma patients with airway hyper-responsiveness to cold and osmotic stimuli. *Bull Siberian Med.* (2017) 16:159–69.

[42] Kato Y. Neutrophil myeloperoxidase and its substrates: formation of specific markers and reactive compounds during inflammation. *J Clin Biochem Nutr.* (2016) 58:99–104. 10.3164/jcbn.15-104 27013775 PMC4788398

[43] Allegra M Tesoriere L Livrea MA. Betanin inhibits the myeloperoxidase/nitrite-induced oxidation of human low-density lipoproteins. *Free Radic Res.* (2007) 41:335–41. 10.1080/10715760601038783 17364963

[44] Tauber E Herouy Y Goetz M Urbanek R Hagel E Koller DY. Assessment of serum myeloperoxidase in children with bronchial asthma. *Allergy.* (1999) 54:177–82. 10221442 10.1034/j.1398-9995.1999.00797.x

[45] Matera MG Calzetta L Gritti G Gallo L Perfetto B Donnarumma G Role of statins and mevalonate pathway on impaired HDAC2 activity induced by oxidative stress in human airway epithelial cells. *Eur J Pharmacol.* (2018) 832:114–9. 10.1016/j.ejphar.2018.05.023 29782855

[46] Chung KF Marwick JA. Molecular mechanisms of oxidative stress in airways and lungs with reference to asthma and chronic obstructive pulmonary disease. *Ann N Y Acad Sci.* (2010) 1203:85–91. 10.1111/j.1749-6632.2010.05600.x 20716288

[47] Barnes PJ. Corticosteroid resistance in patients with asthma and chronic obstructive pulmonary disease. *J Allergy Clin Immunol.* (2013) 131:636–45. 23360759 10.1016/j.jaci.2012.12.1564

[48] Bhavsar P Ahmad T Adcock IM. The role of histone deacetylases in asthma and allergic diseases. *J Allergy Clin Immunol.* (2008) 121:580–4. 18234319 10.1016/j.jaci.2007.12.1156

[49] An TJ Rhee CK Kim JH Lee YR Chon JY Park CK Effects of macrolide and corticosteroid in neutrophilic asthma mouse model. *Tuberc Respir Dis.* (2018) 81:80–7. 10.4046/trd.2017.0108 PMC5771750 29332324

[50] Xia M Xu H Dai W Zhu C Wu L Yan S The role of HDAC2 in cigarette smoke-induced airway inflammation in a murine model of asthma and the effect of intervention with roxithromycin. *J Asthma.* (2018) 55:337–44. 10.1080/02770903.2017.1337788 28960099

[51] Zhou Y Wang GF Yang L Liu F Kang JQ Wang RL Treatment with 1,25(OH)2D3 induced HDAC2 expression and reduced NF-kappaB p65 expression in a rat model of OVA-induced asthma. *Braz J Med Biol Res.* (2015) 48:654–64. 10.1590/1414-431X20154271 25923460 PMC4512106

[52] Xu Y Yunqiu J Changzheng W. Does IL-17 Respond to the Disordered Lung Microbiome and Contribute to the Neutrophilic Phenotype in Asthma? *Mediat Inflamm.* (2016) 2016:1–7. 10.1155/2016/6470364 26941484 PMC4749797

[53] Ricciardolo FL Sorbello V Folino A Gallo F Massaglia GM Favat G Identification of IL-17F/frequent exacerbator endotype in asthma. *J Allergy Clin Immunol.* (2017) 140:395–406. 10.1016/j.jaci.2016.10.034 27931975

[54] Östling J Geest M v Schofield JP Jevnikar Z Wilson S Ward J IL-17-high asthma with features of a psoriasis immunophenotype. *J Allergy Clin Immunol.* (2019) 144:1198–213. 10.1016/j.jaci.2019.03.027 30998987

[55] Hong L Herjan T Bulek K Xiao J Comhair SA Erzurum SC Mechanisms of Corticosteroid Resistance in Type 17 Asthma. *J Immunol.* (2022) 209:1860–9. 36426949 10.4049/jimmunol.2200288 PMC9666330

[56] Nascimento CL Fraga RR de Cássia Rolim Barbosa AL. Effects of Anti-IL-17 on inflammation, remodeling, and oxidative stress in an experimental model of asthma exacerbated by LPS. *Front Immunol.* (2017) 8:1835. 10.3389/fimmu.2017.01835 29379497 PMC5760512

[57] Xie Y Abel PW Casale TB Tu Y. T(H)17 cells and corticosteroid insensitivity in severe asthma. *J Allergy Clin Immunol.* (2022) 149:467–79. 34953791 10.1016/j.jaci.2021.12.769 PMC8821175

[58] Busse WW Holgate S Kerwin E Chon Y Feng J Lin J Randomized, double-blind, placebo-controlled study of brodalumab, a human anti-IL-17 receptor monoclonal antibody, in moderate to severe asthma. *Am J Respir Crit Care Med.* (2013) 188:1294–302. 10.1164/rccm.201212-2318OC 24200404

